# Solution Structure of CXCL5 — A Novel Chemokine and Adipokine Implicated in Inflammation and Obesity

**DOI:** 10.1371/journal.pone.0093228

**Published:** 2014-04-02

**Authors:** Krishna Mohan Sepuru, Krishna Mohan Poluri, Krishna Rajarathnam

**Affiliations:** 1 Department of Biochemistry & Molecular Biology, University of Texas Medical Branch, Galveston, Texas, United States of America; 2 Sealy Center for Structural Biology and Molecular Biophysics, University of Texas Medical Branch, Galveston, Texas, United States of America; National Institute for Medical Research, Medical Research Council, London, United Kingdom

## Abstract

The chemokine CXCL5 is selectively expressed in highly specialized cells such as epithelial type II cells in the lung and white adipose tissue macrophages in muscle, where it mediates diverse functions from combating microbial infections by regulating neutrophil trafficking to promoting obesity by inhibiting insulin signaling. Currently very little is known regarding the structural basis of how CXCL5 mediates its novel functions. Towards this missing knowledge, we have solved the solution structure of the CXCL5 dimer by NMR spectroscopy. CXCL5 is a member of a subset of seven CXCR2-activating chemokines (CAC) that are characterized by the highly conserved ELR motif in the N-terminal tail. The structure shows that CXCL5 adopts the typical chemokine fold, but also reveals several distinct differences in the 30 s loop and N-terminal residues; not surprisingly, crosstalk between N-terminal and 30 s loop residues have been implicated as a major determinant of receptor activity. CAC function also involves binding to highly sulfated glycosaminoglycans (GAG), and the CXCL5 structure reveals a distinct distribution of positively charged residues, suggesting that differences in GAG interactions also influence function. The availability of the structure should now facilitate the design of experiments to better understand the molecular basis of various CXCL5 functions, and also serve as a template for the design of inhibitors for use in a clinical setting.

## Introduction

**Figure 1:**
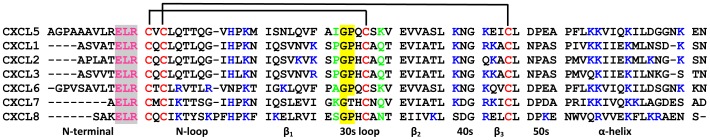
Sequence alignment of CXCR2-activating chemokines. The conserved ELR, cysteine, and GAG-binding residues are highlighted in magenta, red, and blue, respectively. In the 30 s loop region, GP motif and residues that are aligned with I35 and K41 are highlighted in yellow and green, respectively.

Humans express ∼50 chemokines that can be broadly classified on the basis of their conserved cysteines into two major (CXC and CC) and two minor subfamilies (CX3C and C) [1], [2]. These small cytokine-like proteins play crucial roles in defining innate and adaptive immune responses by regulating trafficking of leukocytes, and also play a key role in developmental biology and cancer progression [3], [4]. CXC chemokines can be further divided into sub-classes on the basis of sequence and function. For instance, a subset of seven chemokines (CXCL1, CXCL2, CXCL3, CXCL5, CXCL6, CXCL7, and CXCL8), characterized by the highly conserved ELR motif preceding the N-terminal Cys, selectively activate the CXCR2 receptor (Figure 1) [5].

CXCL5 (also known as ENA78) is expressed in highly specialized cells such as the alveolar epithelial type II cells, white adipose tissue macrophages in the muscle, and cardiomyocytes. This selectivity is particular evident in the lung, as type I epithelial cells and resident macrophages do not express CXCL5, unlike chemokine CXCL8 which is expressed by both lung tissue cells and macrophages [6]. CXCL5 elicits its functions by binding CXCR2 but the outcome is highly context dependent. In the lung, CXCL5 mediates neutrophil trafficking in response to microbial infection by activating both G-protein and arrestin mediated signaling pathways [6], [7]. In the adipose tissue, it functions as an adipokine by activating the Jak2/Stat5 pathway that inhibits insulin signaling and promotes obesity [8], [9]. CXCL5 also activates the mitogen activated protein kinase (MAPK) and phosphoinositide-3 (PI3K) kinase signaling pathways in various cell types [10]. CXCL5-mediated function also involves binding to endothelial and extracellular matrix glycosaminoglycans (GAGs) [11]–[13]. Aberrant CXCL5 levels have been detected in various acute and chronic diseases including microbial infections, rheumatoid arthritis, obesity, chronic pancreatitis, inflammatory bowel diseases, sun burn (UV), and several cancers [14]–[17]. Currently nothing is known regarding the structural basis by which CXCL5 mediates its diverse functions in health and disease. In order to address this missing knowledge, we have solved the solution structure of the CXCL5 dimer using NMR spectroscopy. CXCL5 adopts the typical chemokine fold but also shows several distinct differences in the N-terminal, 30 s loop, and GAG-binding residues. In light of the structure, we discuss how CXCL5-CXCR2 receptor interactions and GAG binding likely mediate diverse functions in various cell types.

## Materials and Methods

### Cloning, expression, and purification

CXCL5 was recombinantly expressed as a fusion protein from the pET32 Xa/LIC plasmid containing the CXCL5 gene inserted at the ligation-independent cloning site. The plasmid was transformed into *Escherichia coli* BL(21)DE3 cells (Invitrogen) and cultured in ^13^C/^15^N-labeled minimal media. Briefly, the transformed cells were grown to an optimal *A*_600_ of 0.6 and induced with 0.2 mM IPTG for 16–20 h at 25°C. The fusion protein from the lysed cells was purified using a nickel-nitrilotriacetic acid column, dialyzed against the cleavage buffer (50 mM Tris, 50 mM sodium chloride, 2 mM calcium chloride, pH 7.4), cleaved using Factor Xa (Novagen, Madison, WI), and CXCL5 was purified using reverse-phase high-performance liquid chromatography. The protein was checked for purity by SDS-PAGE and MALDI-TOF, lyophilized, and stored at −20°C until further use.

### NMR spectroscopy

Chemical shift assignments were achieved using standard 2D and 3D heteronuclear NMR experiments performed at 25°C using a uniformly ^13^C/^15^N-labeled sample (∼0.3–0.5 mM in monomer concentration) in 50 mM sodium phosphate buffer pH 6.0 containing 0.01% sodium azide and 10% D_2_O. The NMR spectra were acquired on a Bruker AVANCE III 800 MHz spectrometer equipped with a TCI cryoprobe. The following 3D NMR experiments were collected for main chain resonance assignments: HNCO, HNCACB, CBCA(CO)NH, HBHA(CO)NH; and following for side-chain assignments: CC(CO)NH, HCC(CO)NH, ^15^N-edited TOCSY-HSQC, ^15^N-edited NOESY-HSQC, and HCCH-TOCSY [18]. The inter-proton NOEs for distance constraints were obtained from 3D ^15^N-edited and ^13^C-edited NOESY. The NMR data were processed using Bruker Topspin and analyzed using Sparky [19].

Native-state Hydrogen Exchange (NHX) studies for identifying slow-exchanging amides and the protection factors of individual NH protons were calculated as described previously [20]. The NHX was initiated by dissolving the lyophilized protein in D_2_O, and a series of HSQC spectra were recorded over a period of 24 hours with each spectrum taking ∼30 mins. ΔG_HX_ is defined as the free energy of unfolding that could be either due to global unfolding or local fluctuations for any given residue, and is given by the equation ΔG_HX_ = −RTlnK_op_, where K_op_ is the equilibrium constant of unfolding [20].

Steady-state {^1^H}-^15^N heteronuclear NOEs were measured using a gradient selected sensitivity-enhanced pulse sequence on a 600-MHz Bruker spectrometer in an interleaved fashion with a proton saturation time of 2.5 s and a relaxation delay of 2.5 s. 200 complex increments and 256 scans/fid were used for signal-averaging the data. Steady-state ^1^H-^15^N NOEs were calculated as a ratio of intensities of the peaks with and without proton saturation. The errors in the NOEs were obtained as described [21].

### Structure calculations

The NOE distance restraints were assessed from ^15^N-edited and ^13^C-edited HSQC-NOESY spectra (mixing time 150 msec). The intensity limits were selected as described for ARIA 2.3, and cross peaks were classified into strong (1.8–2.7 Å), medium (1.8–3.3 Å), weak (2.3–4.5 Å), and very weak (2.8–5.5 Å) [22]. Backbone dihedral restraints were obtained from the chemical shift data using the PREDITOR protocol [23]. The hydrogen-bond (H-bond) restraints of 1.5–2.5 Å for r_HN-O_ and 2.3–3.2 Å for r_N-O_ were applied in the final stages of the refinement. Simulated annealing (SA) calculations with experimental restraints were carried out using the CNSsolve software package [24]. The final 20 structures were chosen on the basis of low total energy, no NOE distance violations >0.25 Å, and no dihedral angle violations >4^o^. The quality of the calculated structures was evaluated using PROCHECK [25] and MOLMOL [26]. The 20 structures have been deposited in the Protein Data Bank (PDB ID code: 2MGS).

## Results

### Characterization of CXCL5 oligomeric state

All CXCR2-activating chemokines studied to date exist reversibly as monomers and dimers and some as tetramers [27]–[30]. Therefore, we characterized the oligomeric state of CXCL5 using NMR and ultracentrifugation studies. The initial NMR HSQC and DOSY and sedimentation velocity experiments at 100 μM in phosphate buffer pH 6.0 indicated CXCL5 exists as a dimer (Figure S1). We therefore measured a series of HSQC spectra at different concentrations (5 to 100 μM) and different pH (5.0 to 7.5), and observed dimer dissociation at lower concentrations and higher pH.

**Figure 2:**
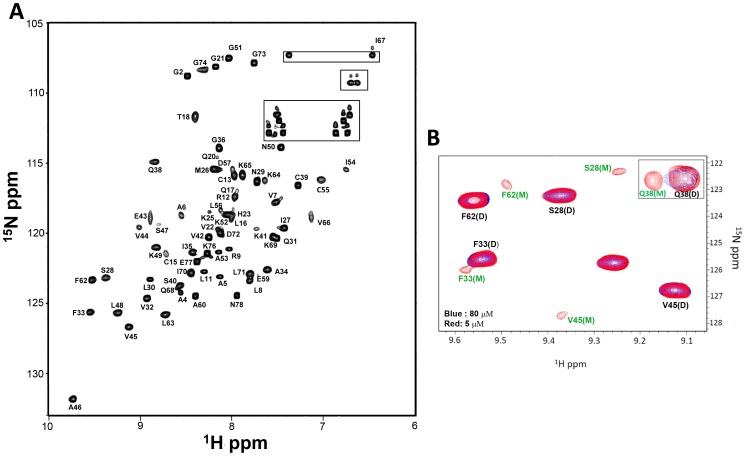
Structural Characterization of the CXCL5 dimer. (A) ^15^N-^1^H HSQC 800 MHz NMR spectrum of the CXCL5 dimer at pH 6.0 and 25°C. The spectrum shows excellent chemical shift dispersion indicating a well-folded single species with no evidence of heterogeneity. The NH2 resonances of asparagines and glutamines are boxed. (B) Sections of the ^15^N-^1^H HSQC spectra at pH 7.5 and 25°C at 5 μM (red) and 80 μM (blue). A new set of peaks corresponding to the monomer is evident in the 5 μM spectrum. The equilibrium constant was calculated to be ∼0.3 μM on the basis of monomer and dimer intensities.

In the HSQC spectra, we could distinguish peaks for both monomers and dimers for Ser28, Phe33, Val45 and Gln38 residues at lower protein concentrations (5, 8, and 10 μM) and pH 7.5 (Figure 2). We calculated the monomer-dimer equilibrium constant (K_d_) for each residue on the basis of the relative intensities of the monomer and dimer peaks at three different concentrations as described previously [31]; The calculated K_d_ varied between 0.2 and 0.5 μM (average 0.3 μM). The dimer dissociation constants of CXCL8, CXCL1, and CXCL7 also vary with solution conditions. For instance, K_d_ for CXCL8 has been shown to vary between ∼0.1 and 14 μM as a function of pH, ionic strength, and buffer [28]–[30].

### Chemical shift assignments

^15^N HSQC spectra collected over 20 to 35°C indicated that the spectrum at 25 °C afforded the best resolution, and so all subsequent NMR experiments were collected at this temperature (Figure 2). The backbone and side chain chemical shift assignments could be achieved for 98% and 95% of residues, and some termini residues could not be assigned unambiguously due to chemical shift degeneracy. The list of NMR chemical shift assignments has been deposited in the BioMagResBank (BMRB accession number: 19601). The chemical shift index (CSI) analysis of the C^α^, C^β^, C′, and H^α^ assignments indicated the presence of three β-strands and one α-helix in a β---β---β---α arrangement (Figure S2). NOE analysis and structure calculations further confirmed the organization of these secondary structural elements.

The C^α^ and C^β^ chemical shifts of the four conserved cysteines (Cys13, Cys15, Cys39, and Cys55) are in the range of 55.8–57.7 and 42.2–45.5 ppm indicating that they are disulfide bonded [32]. A distinctive downfield chemical shift at 10.78 ppm was assigned to the Val14 amide proton. The structure shows that the Val14 amide proton is H-bonded to the side-chain carboxylate of Glu43 (*vide infra*). Similar large downfield chemical shifts for the corresponding residues were also observed for CXCL1, CXCL2, and CXCL8, and structures also reveal that they are H-bonded to a Glu side chain carboxylate [33]–[38].

### Structure of CXCL5

A total of 2250 experimental restraints were used for the final dimer structure calculations. These contain 1796 distance, 264 dihedral (140 φ and 124 φ) and 110 H-bonding constraints for dimer, and 70 distance and 10 H-bonding constraints between the monomers. To generate an initial model of the global fold, a set of 1156 manually assigned and unambiguous NOEs and 176 PREDITOR-derived phi/psi dihedral angle restraints were used. This preliminary model was used to resolve ambiguous NOEs and then added to the restraint set and this method was continued iteratively. In the late stages of refinement, H-bond restraints were added based on H/D exchange data. An ensemble of 200 structures was calculated with the final set of restraints and 20 structures with lowest energy were further analyzed.

**Figure 3:**
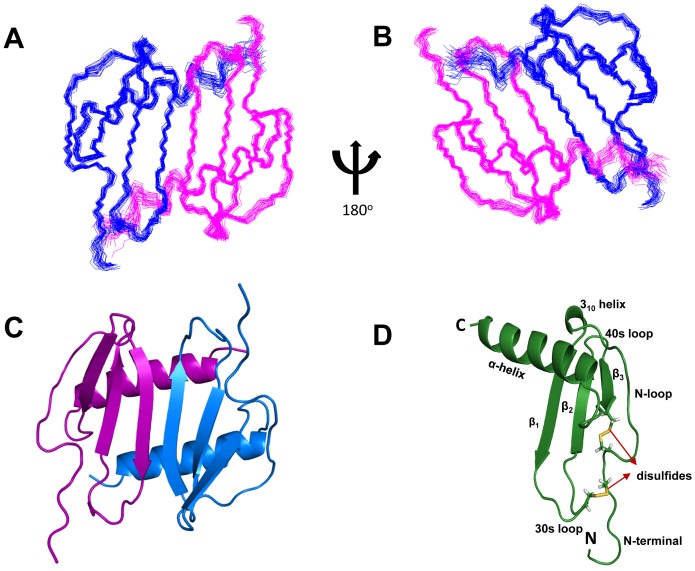
NMR Solution Structure of CXCL5 dimer. Panels A and B show the superposition of the backbone atoms N, Cα and CO of residues 10 to 78 for the calculated twenty structures in two orientations. The polypeptide backbone is colored in blue and magenta for the two monomers. (C) Ribbon representation of the CXCL5 [10–78] structure. The protein dimer comprises of a six-stranded antiparallel β-sheet and a pair of α-helices. (D) Ribbon representation of CXCL5 monomer. The monomer consists of three antiparallel beta strands and an alpha helix; the disulfide bonds are shown in yellow.

The superposition of the individual structures on the average structure is shown in Figure 3. The structure is well defined except for the terminal residues 1–11 and 77–78. The quality of the structures was verified using PROCHECK and VADAR for various criteria such as the stereochemistry, H-bonds, the region of occupancy in the Ramachandran plot, van der Waals contacts, buried charged residues, and packing defects [25], [39]. All 20 structures met criteria expected of a high-resolution structure with none showing NOE violations >0.3 Å and dihedral angle violations >4°. The root mean square distribution (rmsd) for residues 12 to 76 between 20 SA and the average structure is 0.45 Å for the backbone atoms and 1.09 Å for the heavy atoms. The coordinates for the 20 final NMR structures and a list of NMR restraints have been deposited in the Protein Data Bank (PDB accession code: 2MGS).

**Figure 4:**
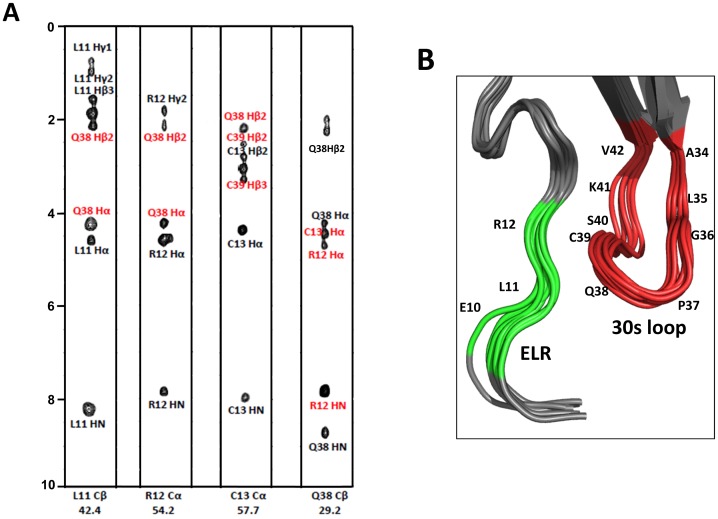
Interactions between 30 s loop and ELR residues. (A) Strip plots of the ^13^C-edited NOESY spectra from L11Cβ, R12Cα, C13Cα, and Q38Cβ. The intra and inter-residue NOEs are labeled in black and red, respectively. (B) Zoomed-in view of the ELR motif (in green) and 30 s loop (in red) residues.

The monomer structure consists of an extended N-terminal loop (N-loop) followed by three β- strands and a terminal α-helix (Figure 3D). The core structure is well defined by a number of long-range hydrophobic contacts between residues of the α-helices and β-strands – Val22 to Ile54/Val66, Pro24 to Val66, Ile27 to Val66/Ile67, Leu30 to Val66/Ile67, Val32 to Ile67, Val44 to Ile67, Leu56 to Ala60/Phe62, and Ala60 to Leu56/Phe62. Besides the disulfide bonds, long-range NOEs between Cys13 and Gln38/Cys39, Val14 and Phe33/Cys39/Glu43/Val45, Cys15 and Cys55, Gln17 and Cys55, Thr18 and Leu56/Asp57, Thr19 and Ile54/Asp57, Gln20 and Phe62/Leu63, Gly21 and Phe62, Val22 and Ile27, Ile54 and Leu56 to Val66, and His23 to Val66 orient the N-terminal loop and the N-terminal residues with respect to the core structure (Figure 4).

The first 11 residues preceding the CXC motif show no or only sequential NOEs, and therefore lack defined structure. The CXC motif is followed by an N-loop that contains a series of turns at Leu16/Gln17, Gln20/Gly21, and His23 that leads into a 3_10_ helix (residues 24–26). The N-loop is well defined but conformationally flexible and is packed against the third β-strand and C-terminal helical residues. The first β-strand (residues 27–33) is connected by a loop that includes a type III turn (labeled as the 30 s loop) to the second β-strand (residues 43–48), which in turn is connected by a type I turn to the third β-strand (residues 53–56). The third strand leads into the helix (residues 61–76) via a type III turn. Residues 77–78 are unstructured, a feature that is consistent with the lack of long range and a few medium NOEs.

The conserved cysteines form disulfide bonds – N-terminal Cys13 with Cys39 that is part of the turn linking first and second β-strands, and N-terminal Cys15 with Cys55 in the third β-strand. The structures reveal that Cys13-Cys39 disulfide is right-handed and the Cys15-Cys55 disulfide is left-handed. Both disulfide linkages are most likely critical for stability and function, as previous studies for CXCL8 and CXCL1 have shown that mutating either of the disulfides results in loss of structural stability and function [36], [40], [41].

**Figure 5:**
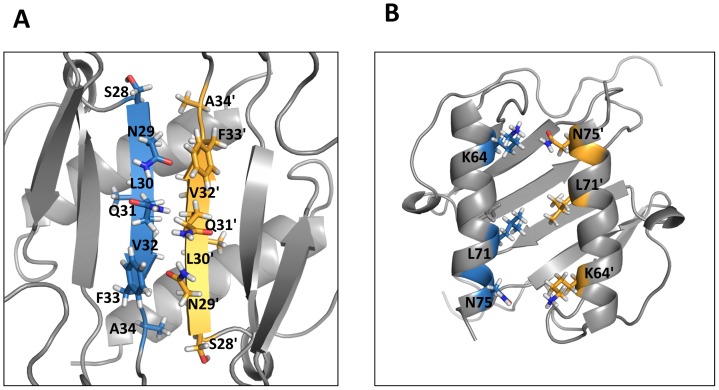
A schematic of dimer-interface interactions. (A) β1/β1′ and (B) α1/α1′ residues that stabilize the dimer interface are highlighted. The interface residues in the monomer units are represented in blue and yellow, respectively.

The ribbon representation of the CXCL5 dimer is shown in Figure 3C. The estimated dimensions of the CXCL5 dimer (excluding side chains) are 33 Å long, 26 Å wide, and 16 Å deep. The structure essentially consists of a six-stranded antiparallel β-sheet with two overlying α-helices. The surface under the β-sheet is composed entirely of hydrophilic and charged residues. The two symmetric helices are ∼23 Å long and separated by a center-to-center distance of 10.2 Å. The angle between the two helices is 168.6°. The dimer interface consists of strand 1 from one monomer and strand 1′ of the second monomer with the C-terminal helix of one monomer interacting with the β-strand of the second monomer. The dimer interface is stabilized by six β-strand backbone H-bonds and a number of packing interactions (Figure 5). The helical residues Ile70 and Gly73 of one monomer are packed against the β-strand residues Ala34 and Val42 of the other monomer, and the β-sheet interface Leu30/Val32, Asn29/Phe33, and Ser28/Ala34 packing interactions further stabilize the dimer-interface. The helical residues Lys64, Ile67, Leu71, and Asn75 are involved in inter-helical interactions across the dimer interface, and in addition, Glu77 is also packed against the 30 s Ile35/Lys41 and the second β-strand Val42.

**Figure 6:**
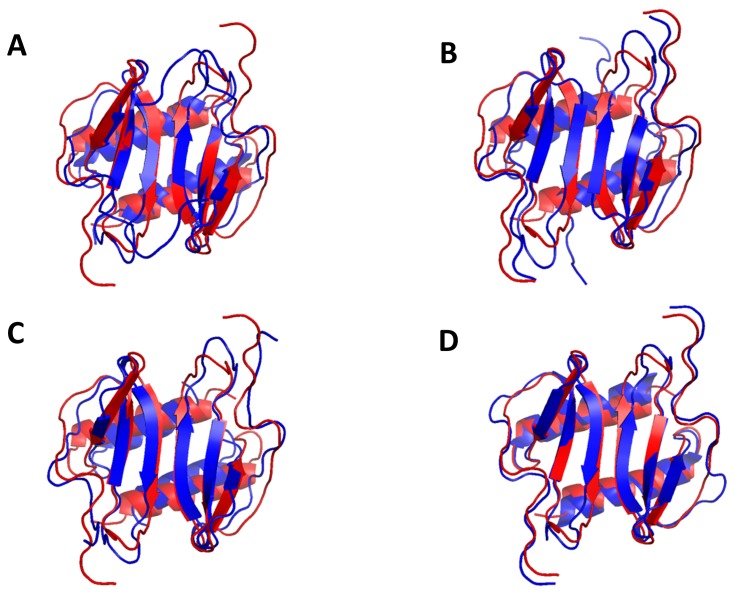
Structural comparison between CXCL5 and other CXCR2-activating chemokines. (A) Superposition of CXCL5 [8–78] (red) on CXCL1, CXCL2, CXCL7, and CXCL8 (blue) in two different orientations. The superposition was optimized using residues 8 to 78 of CXCL5, residues 8 to 73 of CXCL1, residues 6 to 68 of CXCL2, residues 7 to 68 of CXCL7, and residues 8 to 72 of CXCL8.

Overall, the CXCL5 dimer structure is similar to the CXCL1, CXCL2, CXCL7, and CXCL8 dimer structures (Figure 6) [33]–[35], [37], [42]. In particular, the organization of the β-sheet and α-helices is basically identical. Sequence identity between CXCL5 and other CACs vary between 20 to 30% but sequence similarity is higher and ranges between 36 and 51%. Superposition of the backbone residues (from 11 to 76) on other CACs shows rmsd between 1.1 and 2.2 Å. The lowest rmsd is observed for the strands and the helix and higher rmsd for the N-terminal residues, N-terminal loop, and the 30 s turn. Not surprisingly, the latter regions also show the largest sequence differences and are functionally important (Figure 1).

### Stability features of CXCL5

Native state hydrogen/deuterium exchange (NHX) monitored by NMR spectroscopy is a useful tool for defining the H-bonding pattern and also for studying the stabilities of the individual residues [43], [44]. The exchange rates of backbone amide protons depend on accessibility to the solvent deuterons, which in turn correlate with the secondary, tertiary, and quaternary structures, and thermodynamic stability. The NHX data of the CXCL5 dimer show that about half of the resonances disappear within the dead time (8 mins); these residues mostly reside in the N-terminal, N-loop, C-terminal, and interconnecting loop regions of the protein.

**Figure 7:**
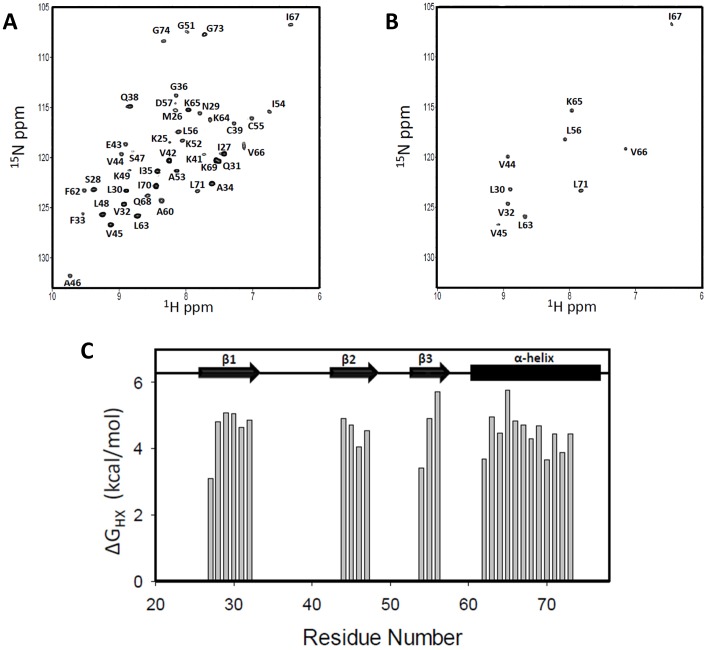
Stability features from amide exchange. HSQC spectra of 100 μM CXCL5 in 50 mM sodium phosphate pH 6.0 after initiating exchange with D_2_O after ∼8 min and after ∼24 hrs. The panel C shows the plot of ΔG_HX_ calculated from the amide exchange data.

The protected residues are predominantly located in the β_1_-, β_2_-, and β_3_-strands and the α-helix. They include β_1_-residues (Ile27, Ser28, Asn29, Leu30, Gln31, and Val32), β_2_-residues (Val44, Val45, Ala46, Ser47), β_3_-residues (Leu54, Cys55, Leu56), and α-helical residues (Phe62, Leu63, Lys64, Lys65, Val66, Ile67, Gln68, Lys69, Ile70, Leu71, Asp72, and Gly73). Sequence analysis reveals that most of these residues are highly conserved indicating their fundamental role in defining the chemokine fold and stability (Figure 1). The HSQC spectra after initiating exchange (∼8 minutes) and after ∼24 hrs are shown in Figures 7A and 7B, respectively. The residue-wise plot of ΔG_HX_ is shown in Figure 7C.

In addition, slow-exchanging amides were also observed for the 30 s loop residues Ile35, Gly36, Cys39, Lys41, and Val42. However, these amides exchange significantly faster compared to the helical and β-sheet amides. The structure reveals that several of these 30s-loop amide protons are either weakly H-bonded or occluded by the steric bulk of the neighboring side chains. In particular, the structure reveals that the amides and carbonyl of Gly36/Cys39 and Ile35/Lys41 are involved in H-bond formation with each other. Ile35/Lys41 in CXCL5 stands out as most CACs have residues with smaller steric bulk such as Ala, Ser, and Pro at 35 and a Gln at position 41. Interestingly, even CXCL6, which shows high similarity to CXCL5, has an Ala at position 35 (Figure 1).

Comparison of the CXCL5 and CXCL8 stability profiles obtained under same experimental conditions also show distinct differences. In CXCL5, the stabilities for all of the β-strand, helical, and dimer-interface residues were essentially similar (Figure 7C). On the other hand, in CXCL8, a number of β-strand residues involved in intramolecular H-bonding interactions were substantially more stable than the dimer interface residues, and also the helical residues showed a range of stabilities [45]. Interestingly, the stabilities of the dimer interface β-strand residues were similar in CXCL5 and CXCL8 suggesting that flexibility of the interface residues might be essential for reversible dimer formation and dissociation. These observations collectively indicate that the dimer stabilities of CXCL5 and CXCL8 are similar but that the stability of the CXCL5 monomer is lower than that of the CXCL8 monomer.

### Backbone dynamics

**Figure 8:**
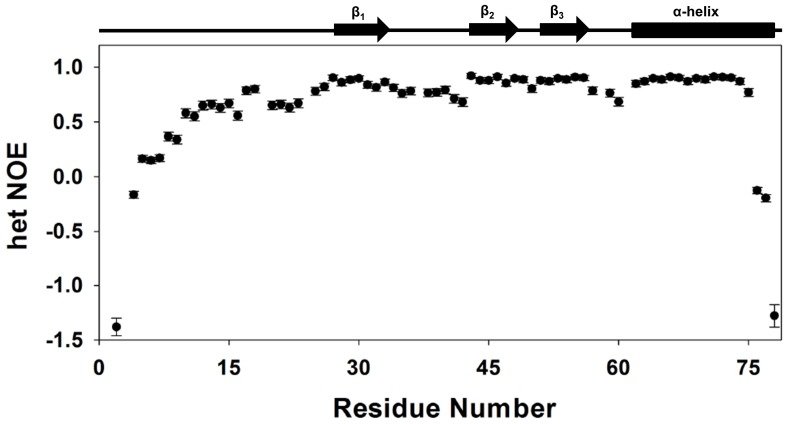
Backbone dynamics. A plot of the backbone {^1^H}-^15^N NOE values as a function of residue number. The data show that the terminal residues are flexible and also the N-loop and the turn residues are more dynamic compared to the helical and strand residues.

Steady state {^1^H}-^15^N heteronuclear NOEs were measured to gain insights into CXCL5's backbone dynamics (Figure 8). Low positive or negative NOE values signify the presence of ns-ps motions. The residue wise plot of the het-NOE indicated ns-ps time scale motions varied substantially among structural elements. The average {^1^H}-^15^N NOE values of β-strand and α-helix backbone amides were calculated to be 0.88, indicating that these structural elements are rigid in the ns-ps time scales. The N-loop that is involved in receptor binding and 30 s loop that indirectly influence receptor binding show lower het-NOE values indicating these regions are more dynamic compared to the core structure. The amide exchange studies also show that the N-loop and 30 s loop residues are conformationally more flexible compared to the β-strand and helical residues (Figure 7).

## Discussion

The chemokine CXCL5 triggers diverse signaling pathways for various functions, including neutrophil trafficking, microbial killing, metabolism, tissue repair, and angiogenesis. The aberrant expression of CXCL5 has been detected in various human diseases including atherosclerosis, inflammatory bowel disease, and cancer [14]–[17]. Though all of CXCL5 functions involve activation of CXCR2, extensive studies over the years have now firmly established that the potency and efficacy of a given chemokine could vary significantly for various CXCR2-mediated activities and different chemokines could show a range of potencies and efficacies for a given CXCR2-mediated activity [46], [47]. Clearly, this information is encoded in the primary sequence, which in turn, determines the structural and functional properties from dimerization and conformational dynamics to affinity, potency, efficacy, and activity. Indeed, sequence analysis reveals both conserved and subtle but distinct differences between CXCL5 and other CACs, suggesting that these residues determine CXCL5's shared and unique functions. We discuss how these residues could determine CXCL5 activity in the context of its tertiary structure and in comparison to what is known about the structures of related CXCR2-activating chemokines.

CXCL5 adopts a structural fold and dimerizes in a manner similar to other CXCR2-activating chemokines [27]–[30]. During active neutrophil recruitment, chemokine concentration could vary by orders of magnitude, and the chemokine could therefore exist as a monomer, dimer, or both at different times and locations. Cellular *in vitro* assays using trapped monomers and dimers of CXCL1 and CXCL8 have shown that the dimer could be as active as the monomer for CXCR2 function, but animal model studies have shown that monomers and dimers are differentially active and that the monomer-dimer equilibrium regulates *in vivo* neutrophil recruitment in a chemokine-specific manner. Chemokine *in vivo* function, in addition to binding CXCR2, also involves binding to highly sulfated glycosaminoglycans (GAGs) in the endothelium and extracellular matrix [11]–[13]. Further, chemokine monomers and dimers show differential GAG binding interactions. On the basis of these observations, we propose that CXCL5 dimer is also likely to be a potent CXCR2 agonist and that its ability to reversibly exist as monomers and dimers regulates *in vivo* function. Studies using trapped monomer and dimer are necessary to provide a more definitive insight into how dimerization mediates CXCL5 *in vivo* function.

CXCR2 receptor activation involves interactions between the ligand N-loop and CXCR2 N-terminal domain residues (defined as Site-I), and between the highly conserved ligand N-terminal ELR residues and the receptor extracellular loops/transmembrane residues (defined as Site-II) [48]–[50]. The structure of CXCR2 is not known, but the structure of the related neutrophil receptor CXCR1 has shown that the N-terminal domain lacks definition and is disordered [51], and it can be assumed that the N-terminal domain in CXCR2 is also unstructured. Our NMR data show that the CXCL5 N-loop residues are structured but conformationally flexible. Therefore, the initial Site-I binding step can be visualized to occur via a fly-casting mechanism with the flexible receptor N-domain capturing the ligand by interacting with the N-loop residues. Sequence analyses show that residues corresponding to Leu16, Thr18, His23, and Lys25 are highly conserved indicating that these residues in CXCL5 also play structural and/or functional roles. Mutating these residues in CXCL1 and CXCL8 impairs activity validating their importance [40], [52], [53]. Because the N-loop sequences of CXCL5 and CXCL1 are largely similar, it can be inferred from CXCL1 binding studies that the CXCL5 N-loop residues from Leu16 to His23 are involved in CXCR2 binding [54]. The CXCL5 structure shows that Thr19 and Gly21 are packed against the 3^rd^ β-strand, Val22 side chain is partially buried, and others are solvent exposed. The buried residues could indirectly influence the binding of the solvent-exposed residues, and/or, because the N-loop is conformationally dynamic, it is also possible that they become transiently exposed and mediate direct binding interactions.

The structural basis of site-II interactions is less well understood, and involves binding of ligand N-terminal residues to receptor residues that are distributed among various extracellular loops and transmembrane helices [55]. The structure reveals that the CXCL5 N-terminal residues that precede the CXC motif are conformationally flexible. The structures of CXCL8, CXCL1, CXCL2, and CXCL7 also show that the N-terminal residues preceding the CXC motif are unstructured [33]–[35], [37], [42], [56], [57]. Mutagenesis and deletion studies in CXCL5 and other CACs have provided compelling evidence that the ELR residues are essential for receptor activity [48], [53], [58], [59]. Sequence analyses show low identity among residues preceding the ELR motif and also indicate variations in the number of residues. Interestingly, the N-terminal tail in CXCL5 is longer, and mutating the arginine residue preceding the ELR motif results in reduced receptor function [58]. The requirement for an additional residue provides further evidence that additional interactions in CXCL5, beside the ELR motif, mediate Site-II interactions [59]. Indeed, *in vivo* proteases cleave the N-terminal tail residues to generate truncation mutants, and a 71-residue variant shows significantly higher activity [59], [60], suggesting that different forms of CXCL5 may play specific *in vivo* roles.

A recent study from our lab has shown that the 30 s loop residues mediate crosstalk between Site-I and Site-II and dictate the distribution of the conformation substates that may be required for the repertoire of CXCR2-mediated functions [50]. In addition to the conserved carboxylate (Glu43) and the GP motif, CXCL5 structure shows the additional feature of a number of slow exchanging amides among the 30 s loop residues. We propose that these structural features in the 30 s loop residues could influence the distribution of the conformational substates in CXCL5, which could explain the activation of various downstream signaling pathways including Jak2/Stat2 pathway in the adipose tissue.

**Figure 9:**
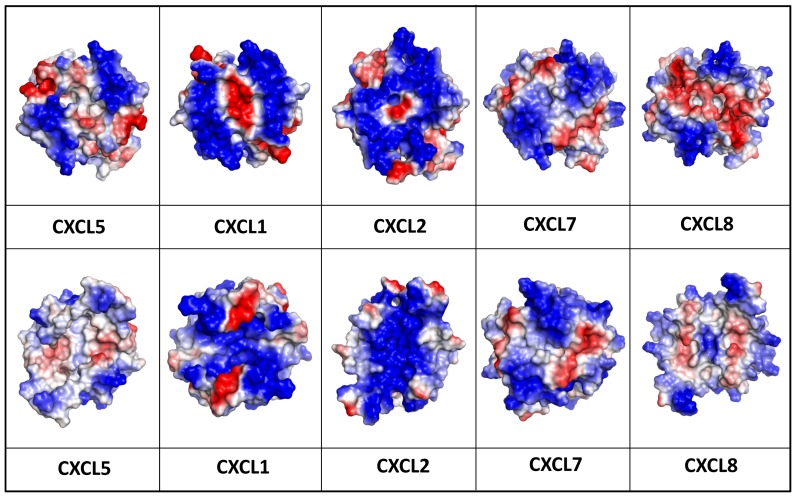
Electrostatic representation of CXCR2-activating chemokines. The upper panels show the helical surface that highlight distribution of the GAG-binding residues and the lower panels show the opposite β-sheet surface after a 180° flip.

CAC function also involves binding to endothelial and extracellular matrix GAGs that allow establishing haptotactic (surface) and chemotactic (soluble) gradients, a process that plays a pivotal role in cellular trafficking [61], [62]. Mutagenesis studies in CXCL8 and mouse CXCL1 have shown that electrostatic interactions mediate chemokine-GAG interactions, and a subset of positively charged Lys/Arg/His residues in the C-terminal helix and the proximal loop mediate GAG binding [63], [64]. Computer modeling, NMR, and *in vivo* studies have also shown that the role of the GAG-binding residues are not equivalent, and each residue could play differential roles in determining affinity, specificity, and geometry of binding. Sequence analyses show that many (including Lys64, Lys65, and Lys69 from the helix and His23 and Lys25 from the proximal loop) but not all of these residues are conserved in CXCL5 (Figure 1). Indeed, the CXCL5 structure reveals that the surface charge distribution is distinctly different compared to other CACs suggesting differences in GAG interactions and hence function (Figure 9). Future mutagenesis studies should provide more definitive structural insights into how GAG interactions influence *in vivo* recruitment.

In summary, the solution structure of CXCL5 dimer reveals a well-defined core consisting of β-strands and helices, a typical chemokine fold, and dimerization similar to other CXCR2-activating chemokines. The structure nevertheless also reveals that the local structure and dynamics of the 30 s loop residues are distinct, that the N-terminal residues are unstructured despite the relatively longer size, and these differences most likely dictate CXCR2-signaling and various activities. The structure also reveals that the surface charge distribution of GAG-binding residues is different. We propose that these structural features define CXCL5 function, and that the structure will facilitate future studies aimed at achieving a better understanding of the underlying molecular mechanisms, and the design of structure-based decoys for various CXCL5-mediated diseases.

## Supporting Information

Figure S1 (A) Translational self-diffusion coefficient measurement of CXCL5. Non-linear least squares fitting of the normalized intensity data for a side chain methyl resonance at ∼0.9 ppm of a 100 μM sample obtained by varying gradient strength. The NMR self-diffusion coefficients (D_S_) were measured using a stimulated echo and LED incorporating bipolar gradient pulses for diffusion [45]. (B) A schematic of the sedimentation velocity profile showing CXCL5 at 100 μM is a dimer. Sedimentation velocity studies were performed using a Beckman-Coulter Optima XL-A analytical ultracentrifuge equipped with absorbance optics and a Ti-60a titanium rotor. 100 μM protein samples in 50 mM phosphate, 50 mM NaCl (pH 6.0) were centrifuged at 45,000 rpm at 25°C. The protein absorbance was measured at 215 nm, and data were analyzed using Hetero-Analysis software version 1.1.33 (J. L. Cole and J. W. Larry, University of Connecticut).
(TIF)

Figure S2 **Chemical shift index (CSI) plot derived using Hα, Cα, Cβ, CO chemical shift deviations from random coil values defining elements of secondary structure as a function of residue number.**
(TIF)
