# Supplementary figures and images for: Solution Structure of CXCL5 — A Novel Chemokine and Adipokine Implicated in Inflammation and Obesity
Source: PLoS One. 2014 Apr 2;9(4):e93228. doi: 10.1371/journal.pone.0093228 (PMC3973705; doi:10.1371/journal.pone.0093228)

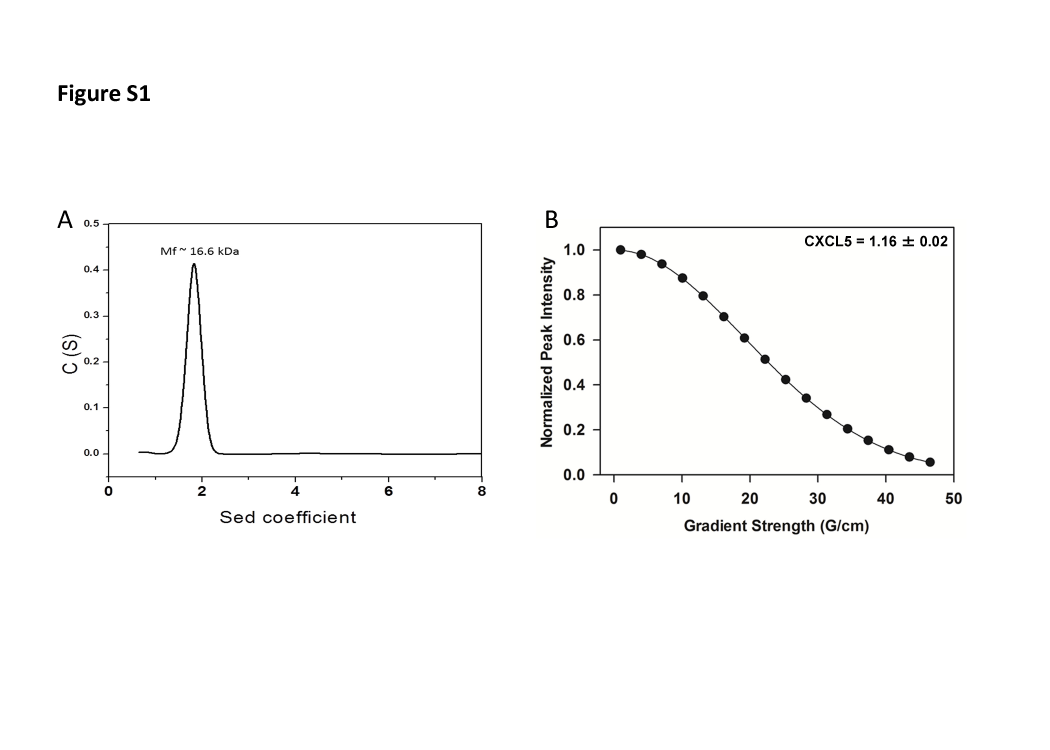

Supplement: Figure S1 — (A) Translational self-diffusion coefficient measurement of CXCL5. Non-linear least squares fitting of the normalized intensity data for a side chain methyl resonance at ∼0.9 ppm of a 100 μM sample obtained by varying gradient strength. The NMR self-diffusion coefficients (DS) were measured using a stimulated echo and LED incorporating bipolar gradient pulses for diffusion [45]. (B) A schematic of the sedimentation velocity profile showing CXCL5 at 100 μM is a dimer. Sedimentation velocity studies were performed using a Beckman-Coulter Optima XL-A analytical ultracentrifuge equipped with absorbance optics and a Ti-60a titanium rotor. 100 μM protein samples in 50 mM phosphate, 50 mM NaCl (pH 6.0) were centrifuged at 45,000 rpm at 25°C. The protein absorbance was measured at 215 nm, and data were analyzed using Hetero-Analysis software version 1.1.33 (J. L. Cole and J. W. Larry, University of Connecticut). (TIF) [file pone.0093228.s001.tif]

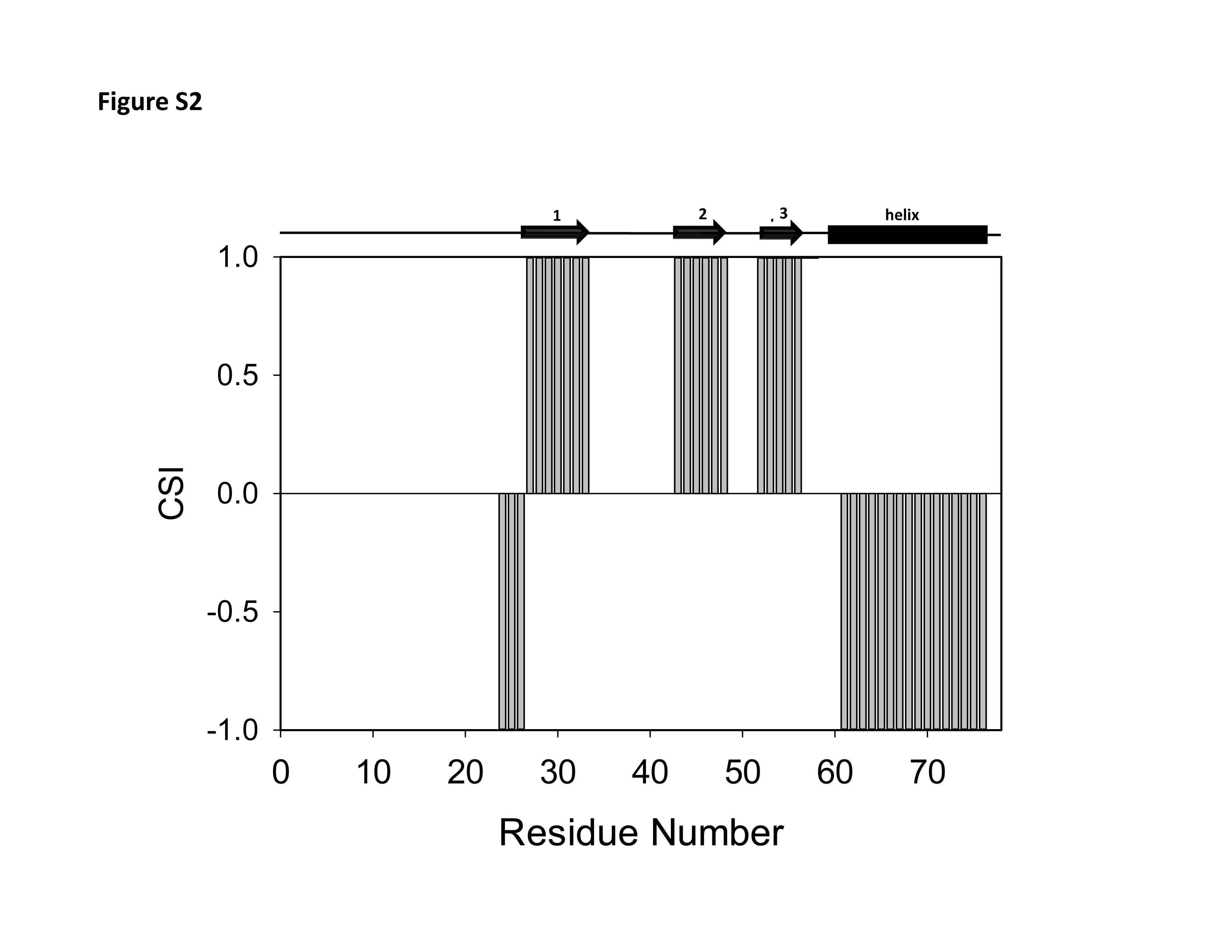

Supplement: Figure S2 — Chemical shift index (CSI) plot derived using Hα, Cα, Cβ, CO chemical shift deviations from random coil values defining elements of secondary structure as a function of residue number. (TIF) [file pone.0093228.s002.tif]
